# Correspondence: DNA shape is insufficient to explain binding

**DOI:** 10.1038/ncomms15643

**Published:** 2017-06-05

**Authors:** Matthew J. Rossi, William K.M. Lai, B. Franklin Pugh

**Affiliations:** 1 Department of Biochemistry and Molecular Biology, Center for Eukaryotic Gene Regulation, The Pennsylvania State University, University Park, Pennsylvania 16802, USA

Nature Communications **8**:15643 doi: ; DOI: 10.1038/ncomms15643 (2017); Published 06 05 2017PMC546534928580956

Proteins bind DNA through combinations of DNA base and shape recognition^1^. DNA base recognition refers to a unique arrangement of protein interactions with functional groups on the four DNA bases. Shape recognition refers to protein interactions with specific twists and turns of short stretches of DNA that may deviate from the average three-dimensional shape of B-form DNA. A recent study by Zentner *et al*.^2^ characterized the genome-wide binding of *S. cerevisiae* DNA binding proteins Abf1, Rap1 and Reb1, reporting many thousands of novel, low-scoring binding sites that lacked a consensus motif sequence. The sites were deemed significant because they reportedly possessed a DNA shape that was highly similar to that of the protein's cognate sites and very different from random sites. We show here that when random sites are processed in precisely the same manner as high- and low-scoring sites, including using a 50 bp search window (which by error was not done in Zentner *et al*.^2^), the low-scoring sites were no different than random, thereby invalidating the applicable conclusions. Since other analyses on slow sites were interpreted based on these invalid conclusions, we find an overall lack of evidence supporting the conclusion that Abf1, Rap1 and Reb1 predominately read DNA shape to recognize thousands of novel ‘low-scoring' sites.

In Figure 7 of Zentner *et al*.,^2^ it was reported that the DNA shape^3^ of low-scoring sites was on average highly similar to the DNA shapes associated with high-scoring sites and significantly different from random sites. From this result, it was concluded that the favourable DNA shape recognition at low-scoring sites captures transient scanning interactions. The analysis used a 50 bp search space centred over high- and low-scoring ChEC-seq (chromatin endogenous cleavage with high-throughput sequencing) peak midpoints, so as to find the best match to a previously published consensus motif^4^. The *P* value threshold was set such that up to three mismatches to the consensus motif were allowed. From this, a DNA shape analysis was performed and compared across sites. We repeated the analysis and obtained precisely the same results for high- and low-scoring sites (Fig. 1a, red versus blue traces). Most critically, Zentner *et al*.^2^ further reported that the same number of random sites, as a negative control, had on average no particular shape property. However, when we repeated this control^3^, we obtained a shape pattern that was essentially no different from the putative low-scoring sites (Fig. 1a, green versus blue traces). If we excluded the 50 bp search (that is, performed a 1 bp search), then we obtained the pattern reported by Zentner *et al*.^2^ (Fig. 1a, black traces). Our reanalysis shows that average DNA shape at low-scoring sites is indistinguishable from random if the best motif is sought equivalently in both data sets. Therefore, this DNA shape analysis^3^ provides no evidence of shape specificity at putative low-scoring sites, and no evidence that ChEC-seq peaks at low-scoring sites are a product of specific DNA shape recognition.

To understand the source of ChEC-seq peaks at putative low-scoring sites, we next investigated the spatial relationship between high-scoring and low-scoring sites. We found that low-scoring sites were physically closer to high-scoring sites in the genome compared to random sites (Fig. 1b). Of the low-scoring sites, ∼50% were within 250 bp of a high-scoring site (compared to ∼8% of random sites), indicating that these low-scoring sites generally exist within the same nucleosome-depleted region (NDRs) as high-scoring sites. Consistent with this, Zentner *et al*.^2^ reported that AT-rich sequences, which are a well-known property of NDRs, are enriched at low-scoring sites. Since micrococcal nuclease (MNase) preferentially cleaves DNA in NDRs^5^, we surmise that the ChEC-seq peaks associated with putative low-scoring sites arise mostly from non-specific MNase cleavage events that are near high-scoring sites in accessible chromatin, perhaps after cleavage release from high-scoring sites. This is in accord with their temporally slow appearance (Fig. 3 in Zentner *et al*.^2^). Since the high-scoring sites for Abf1, Rap1 and Reb1 do not appreciably overlap, it follows that the positionally linked low-scoring sites would also not overlap, nor overlap with an untargeted MNase-only ‘negative' control as reported in Zentner *et al*.^2^

Figure 4 in Zentner *et al*.^2^ reports the distribution of Abf1 X-ChIP-seq (chromatin immunoprecipitation with high-throughput sequencing) peaks around low-scoring sites/motifs. We note a local minimum directly at these motifs, and local maxima ∼50–100 bp away. These observations are consistent with high-scoring sites, which X-ChIP-seq is measuring, being physically close to but not coincident with putative low-scoring sites. Figure 5 in Zentner *et al*.^2^ reports MNase-derived ‘footprints' at high-scoring sites. However, similar footprints are not evident at the putative low-scoring sites. The lack of appreciable binding of factors at putative low-scoring sites using a variety of ChIP assays, as noted in Zentner *et al*.,^2^ provides further evidence that such putative sites are false.

Our analysis refutes the validity of ChEC-seq measurements of Abf1, Rap1 and Reb1 interactions at low-scoring sites, which were reported to occur at ∼10 times the frequency of such locations as measured by ChIP methods. Subsequent efforts to validate a small subset of these slow sites, corresponding to about 12%, 4% and 1% of the original Abf1, Reb1 and Rap1 sites (https://github.com/sivakasinathan/shape_align), respectively, revealed no significant shape feature at the alignment point (Fig. 1c and data not shown). However, ∼50 bp away there was enrichment of A/T (for example, poly(dA:dT)), which is too far away to be a part of the sites. Moreover, their DNA shapes were well-correlated (Fig. 1d), which would not be expected if they were part of the distinctly different shapes of Abf1/Reb1/Rap1 sites. ‘Control' random sites (Fig. 1d) behaved distinctly different because they are not found in A/T-rich NDRs, where putative slow sites are found. Thus, any analysis concluding that ‘slow sites with shape features similar to fast sites are likely true binding sites' is therefore faulty. Measured interactions at high-scoring sites are not questioned.

## Methods

The random sites for each factor were generated using the bedtools^6^ random function, and then filtered to remove overlap with existing ChEC-seq peaks and overlap with each other. The underlying sequence in a 50 bp window around the random sites was then used to search for the best sequence match, using a normalized log-likelihood scoring metric^7^, to motifs for Reb1, Rap1 and Abf1 using consensus motifs from ScerTF^4^. The best match was then used to realign the random regions. DNA shape analysis^3^ was performed on the existing high- and low-scoring ChEC-seq peaks along with the aligned and unaligned random regions.

## Additional information

**How to cite this article:** Rossi, M. J. *et al*. Correspondence: DNA shape is insufficient to explain binding. *Nat. Commun.* **8**, 15643 doi: 10.1038/ncomms15643 (2017).

**Publisher's note:** Springer Nature remains neutral with regard to jurisdictional claims in published maps and institutional affiliations.

## Figures and Tables

**Figure 1:**
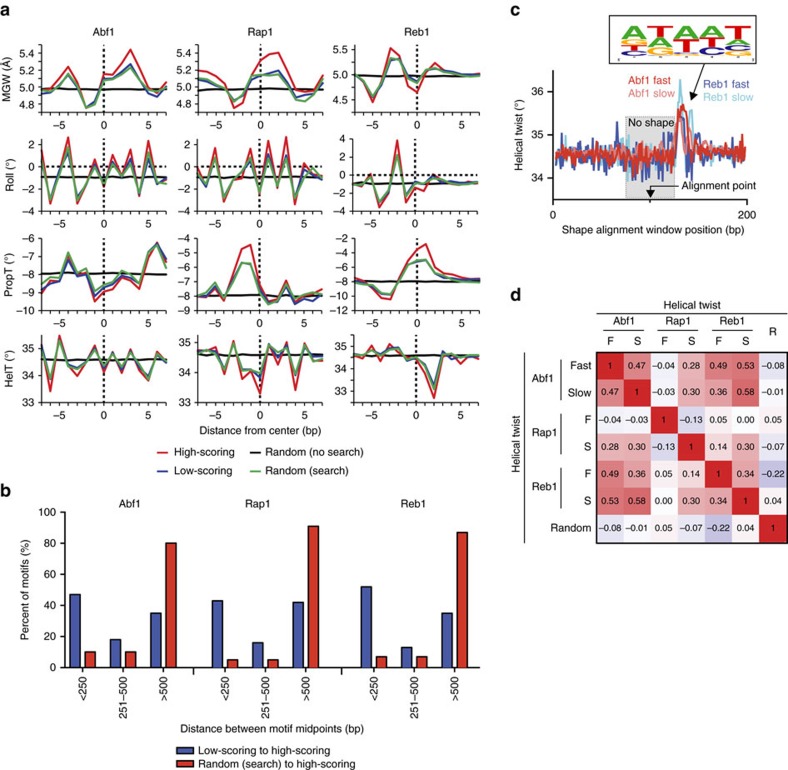
Transcription factor motif-specific DNA shapes are not enriched at low scoring sites. (**a**) When the same search window is allowed to define the reference point for low-scoring (blue trace) and random (green trace) sites, the DNA shape patterns are indistinguishable from each other, unless no search window is employed (black trace). (**b**) The bp distance of each low-scoring site to the closest high-scoring site (blue bars) compared to the distance of each random site to the closest high-scoring site (red bars) for each factor. (**c**) Composite profiles of the aligned helical twist for Abf1 and Reb1 fast and slow sites. Other tested shape parameters behaved similarly (not shown). The frequency web logo is composed of all pentamers that possess an intrinsic helical twist larger than 35.5°. (**d**) Pearson correlations of the composite-aligned helical twist vectors for Abf1, Rap1 and Reb1 fast and slow, and random sites.
